# A Case of Lytic Bone Lesions and Unexpected Laboratory Findings: Navigating Diagnostic Uncertainty

**DOI:** 10.7759/cureus.106004

**Published:** 2026-03-27

**Authors:** Om Jhaveri, John Wahidy, James Byrd

**Affiliations:** 1 Medicine, Alabama College of Osteopathic Medicine, Dothan, USA; 2 Research, Alabama College of Osteopathic Medicine, Dothan, USA; 3 Internal Medicine, Mobile Infirmary Medical Center, Mobile, USA

**Keywords:** art of diagnosis, diagnosis of multiple myeloma, kappa light chain, lambda light chain, light-chain multiple myeloma, monoclonal gammopathy, mr imaging, mr imaging multiple myeloma, multiple myeloma

## Abstract

We present a case of a 66-year-old male patient who presented with progressive generalized weakness and was found to have diffuse lytic lesions involving the calvarium and entire spine. Diffuse lytic bone lesions identified on imaging prompted an extensive evaluation for malignant and non-malignant etiologies. In cases with atypical laboratory findings and negative bone marrow biopsy, diagnosis and management can be complicated. Imaging was initially highly suspicious of malignancy, but further investigation revealed abnormal serum free light chain levels. Additionally, a negative serum and urine protein electrophoresis and bone biopsy revealed no evidence of malignancy or plasma cell dyscrasia. This unique case highlights the diagnostic difficulty and importance of interpretation and lateral surveillance in patients with suspected plasma cell dyscrasia.

## Introduction

Multiple myeloma is a plasma cell malignancy, characterized by clonal proliferation of plasma cells, which crowd out other healthy erythropoietic cells. These neoplastic cells produce monoclonal immunoglobulins (M proteins) and/or light chains. It accounts for approximately 10% of hematologic malignancies and is typically seen in older adults. It presents with the characteristic quartet of hypercalcemia, renal dysfunction, anemia, bone pain, and lytic bone lesions, which is classically summarized as the CRAB criteria [1].

Skeletal involvement is a defining feature of multiple myeloma, as the osteolytic lesions are a product of increased osteoclast activity paired with suppressed osteoblastic function mediated by malignant plasma cells [2]. Diffuse lytic lesions involving the axial skeleton, specifically the skull and vertebral bodies, are classically considered highly suggestive of multiple myeloma or metastatic disease. Consequently, these imaging findings prompt aggressive diagnostic evaluation and urgent therapeutic intervention.

In rare instances, the diagnosis of multiple myeloma can pose a challenge when patients present with atypical clinical features and laboratory findings. The classic CRAB findings may be absent in a particular subset of the population, especially those with early or indolent disease [3]. Additionally, serum electrophoresis (SPEP) may fail to detect a monoclonal protein (M spike) in cases of light-chain-only disease, non-secretory myeloma, or early plasma cell dyscrasias [4]. In these circumstances, serum-free light chain assays are a diagnostic option that can provide critical information [5].

The gold standard for diagnosing plasma cell disorders, such as multiple myeloma, is a bone marrow biopsy, which would show over 10% of irregular plasma cells and often also shows a stacked or “rouleaux” appearance of red blood cells [6]. Bone marrow involvement may be patchy and can often result in false-negative results when sampling is limited to a single site such as the iliac crest. As a result, clinicians may experience diagnostic uncertainty when weighing malignancy, lab results, and histopathological findings.

We present a case of a patient with a diffuse lytic bone lesion with a markedly abnormal serum free light chain ratio. Extensive evaluation, including serum and urine electrophoresis, cross-sectional imaging, and bone biopsy, all failed to establish a definitive diagnosis. This case highlights the limitations of conventional diagnostic criteria and underscores the importance of longitudinal surveillance in patients with suspected plasma cell disorders who present with diagnostic uncertainty.

## Case presentation

A 66-year-old male patient with a medical history significant for hypertension, coronary artery disease, atrial fibrillation on anticoagulation, type 2 diabetes mellitus with stage 3 chronic kidney disease, hyperlipidemia, obstructive sleep apnea, and a prior cerebrovascular accident presented with progressive generalized weakness.

The patient had previously been discharged one week prior, following a left heart catheterization with recommendations for medical management. History from the patient and his spouse revealed that he had been gradually experiencing worsening weakness over approximately six months, culminating in an episode on September 3, where he was unable to exit from a car without assistance, which was an unprecedented functional decline. He denied any focal neurological deficits or any bone pain. During this hospital admission, his symptoms included a dry cough, headache, and shortness of breath for which he tested positive for SARS-CoV-2 on September 4 and was treated with remdesivir.

Imaging findings

Computed tomography (CT) of the brain performed on September 3 demonstrated a subacute to chronic infarction of the right caudate nucleus, along with chronic small vessel ischemic changes and cerebral volume loss, which is consistent with this prior cerebral vascular accident. Multiple lytic lesions were also identified within the calvarium, raising concerns for multiple myeloma or metastatic disease. 

**Figure 1:**
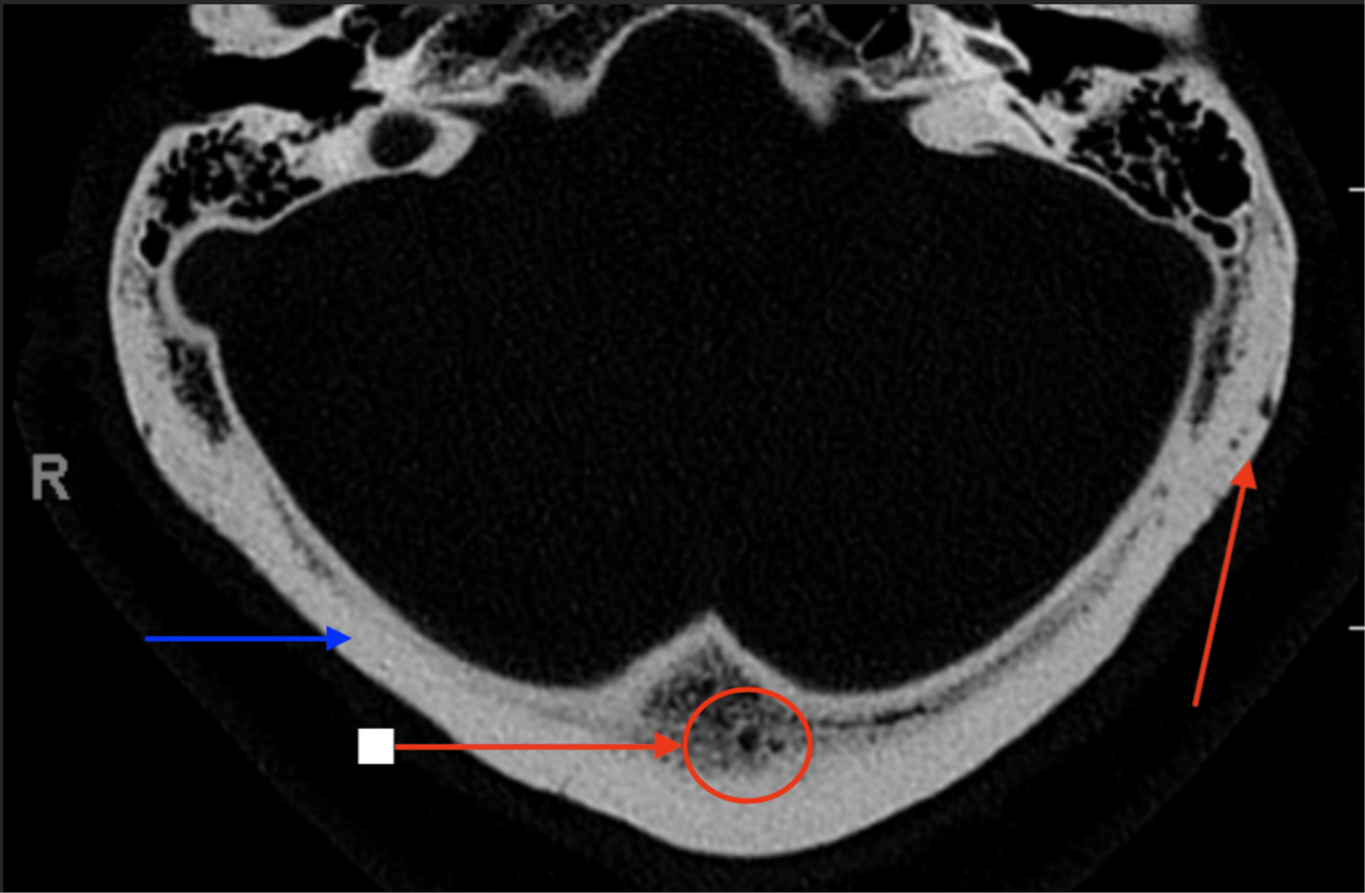
Axial CT of the calvarium showing lytic lesions

**Figure 2:**
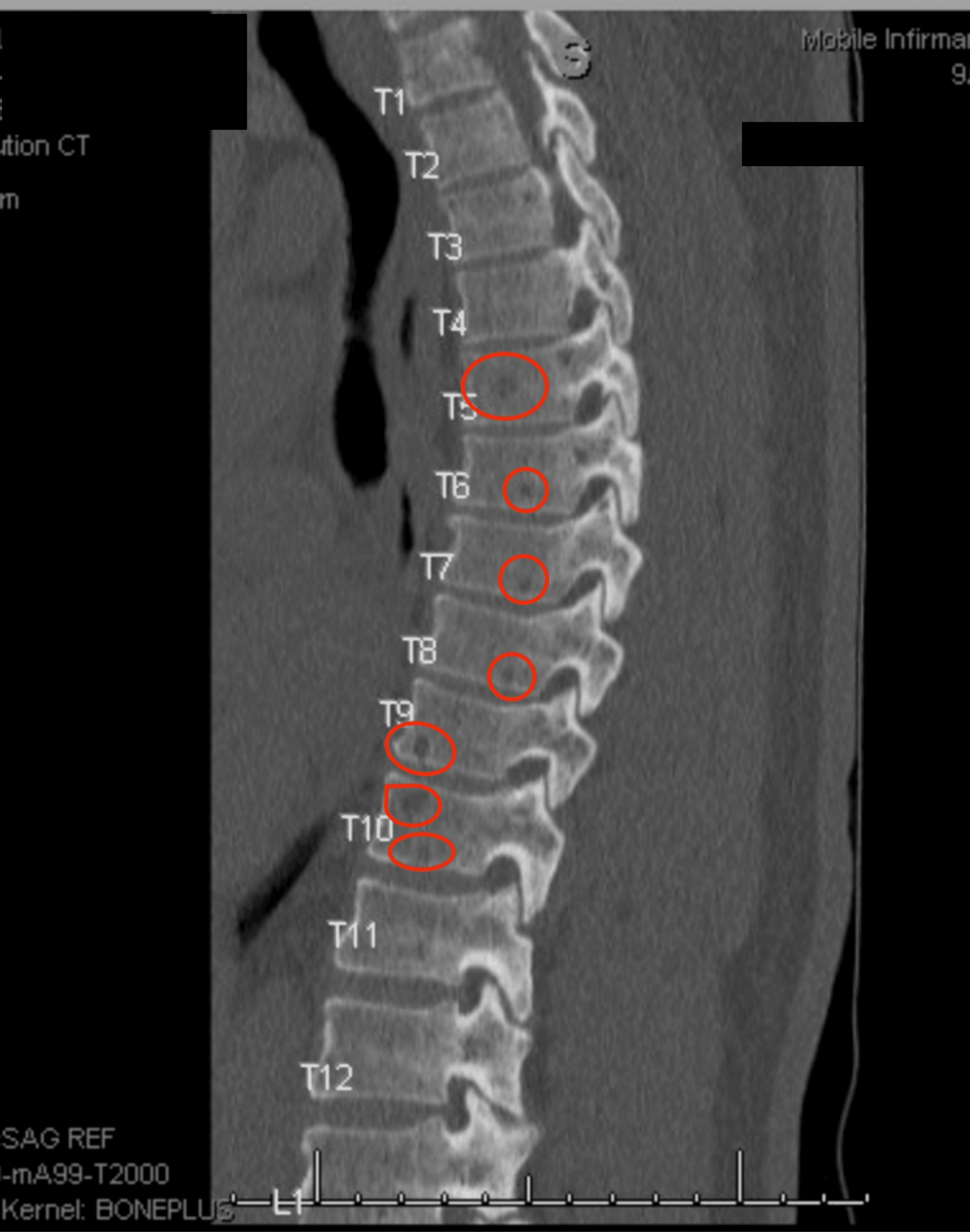
Sagittal CT of the thoracic spine showing multiple lytic lesions, without compression fractures, subluxation, or any paraspinal masses

**Figure 3:**
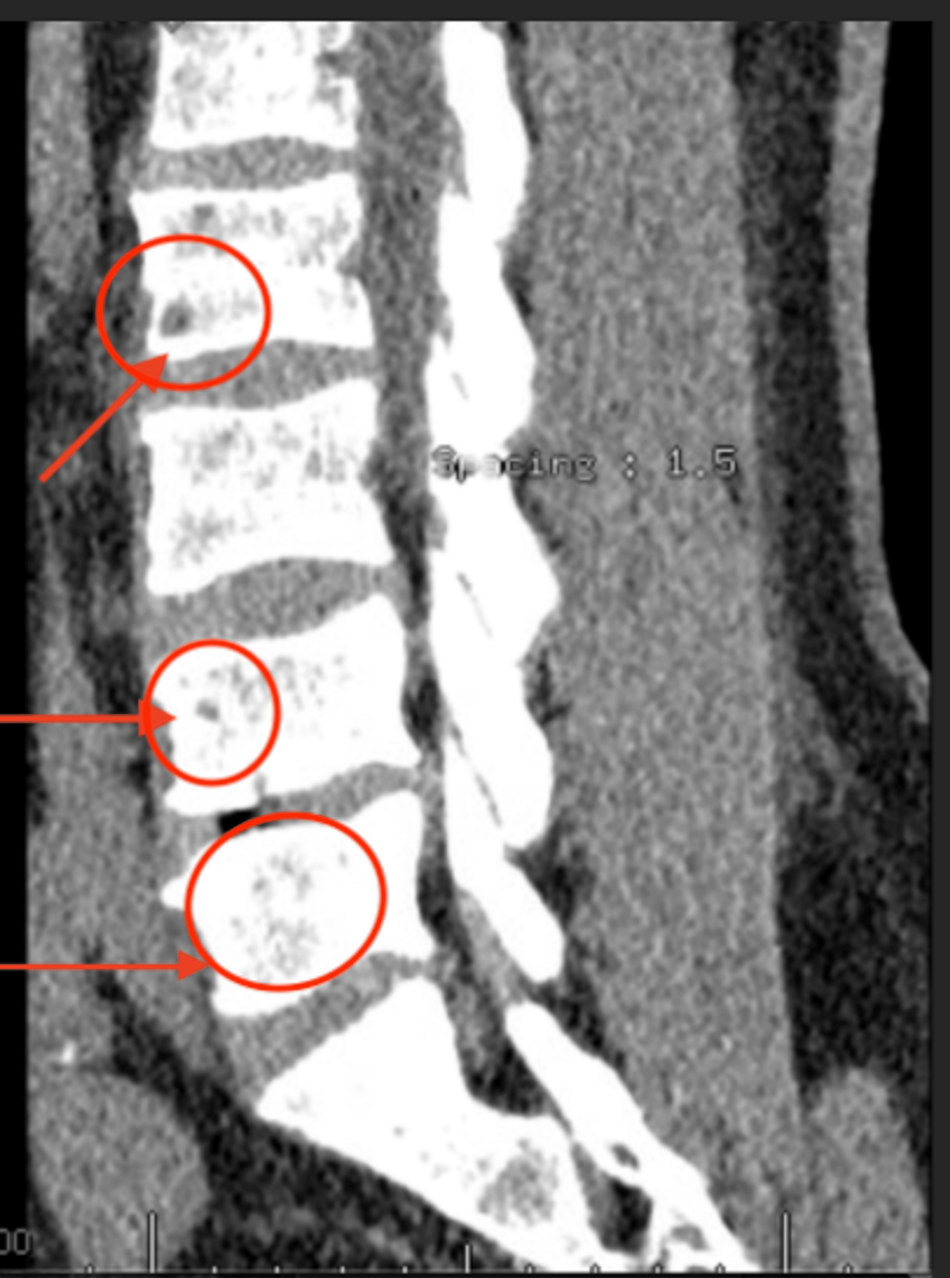
Sagittal CT of the lumbar spine showing SI joint fusion as well as multiple lytic lesions

CT imaging of the calvarium (Figure 1) and thoracic and lumbar spine revealed multiple lytic and small lytic lesions diffusely involving the vertebral bodies, which were also worrisome for metastatic disease or multiple myeloma. CT of the thoracic spine without compression fractures, subluxation, or any paraspinal masses (Figure 2). CT of the lumbar spine additionally showed degenerative changes, including disc bulging from L1 to S1, facet arthropathy, and fusion of the sacroiliac joints (Figure 3). There was also mild spinal canal encroachment from L4 to L5 without significant cord or nerve root compression. Contrast-enhanced CT of the chest, abdomen, and pelvis revealed multiple lytic bone lesions without evidence of a primary malignancy as well. Lesions measured approximately 5-12 mm and were distributed throughout the axial skeleton. Representative lesions are demonstrated in Figures 1-3.

Laboratory evaluation

Initial laboratory studies showed persistent erythrocytosis without anemia. The hematologic studies remained stable throughout the hospitalization. Serum calcium levels were low to mildly decreased on serial measurements. Renal function was consistent with his baseline chronic kidney disease without any evidence of acute renal deterioration. Prostate-specific antigen was within normal limits, and chest radiography was otherwise unremarkable. Serum protein electrophoresis and urine protein electrophoresis demonstrated no monoclonal protein spike. Serum and urine immunofixation electrophoresis (IFE) were performed, with no monoclonal protein detected.

Additionally, urine immunofixation was performed and was negative as well. However, serum free light chain analysis revealed significantly elevated kappa free light chains and an increased kappa-to-lambda ratio, raising concern for a plasma cell disorder despite unremarkable electrophoresis studies. Key laboratory findings are summarized in Table 1.

**Table 1:** Summary of key laboratory findings

Laboratory Test	Patient Value	Reference Range
Hemoglobin	16.5-17.3 g/dl	13.5–17.5 g/dL
Red Blood Cell Count	6.08-5.79 ×10⁶/µL	4.32–5.72 ×10⁶/µL
Hematocrit	54.32-50.1%	38.8–50%
Serum Calcium	8.2-8.7 mg/dL	8.6–10.2 mg/dL
Serum Creatinine	1.59-1.44 mg/dL	0.7–1.3 mg/dL
Estimated Glomerular Filtration Rate (eGFR)	50–54 mL/min/1.73 m²	≥60 mL/min/1.73 m²
Serum Protein Electrophoresis	No M spike detected	No monoclonal protein
Urine Protein Electrophoresis	No M spike detected	No monoclonal protein
Kappa Free Light Chain	220.2 mg/L	3.3–19.4 mg/L
Lambda Free Light Chain	19.4 mg/L	5.7–26.3 mg/L
Kappa/Lambda Ratio	11.35	0.26–1.65
Prostate-Specific Antigen (PSA)	2.40 ng/mL	<4.0 ng/mL

Specialist evaluation and follow-up

For further evaluation, the patient was referred to oncology. Due to the diffuse lytic bone lesions and abnormal free light chain ratio in the absence of classic multiple myeloma features, a CT-guided bone biopsy of the left posterior iliac crest was performed. Histopathology demonstrated benign, normocellular bone without evidence of malignancy or plasma cell dyscrasia.

At the patient's oncology follow-up, he reported no bone pain, weight loss, or constitutional symptoms. Aside from the one episode, he described a nine-month history of gait instability and balance difficulty, which had since then remained stable, requiring a cane for ambulation and intermittent wheelchair use. Further imaging with MRI of the lumbar spine showed no significant spinal cord or nerve root compression. Given his history of prior cerebral vascular accident and possible additional undocumented events, a neurology referral was also arranged. In the absence of a definitive diagnosis, the oncologist recommended close surveillance with interval follow-up and instructed him to report any new bone pain, weight loss, or systemic symptoms.

## Discussion

This case demonstrates navigating a challenging diagnostic scenario in which radiographic findings were highly suggestive of a specific pathology, yet laboratory and histopathologic data failed to confirm it [7]. The presence of diffuse lytic lesions involving the calvarium and vertebral bodies is classically associated with multiple myeloma or metastatic disease, and such findings prompt extensive malignancy workup [8,9].

Despite the concerning radiographic findings, the patient lacked several hallmark features of multiple myeloma. Notable findings per CRAB criteria [10] that weren’t demonstrated include persistent erythrocytosis rather than anemia. The presence of erythrocytosis is atypical for multiple myeloma and further contributed to diagnostic uncertainty. This patient’s persistent erythrocytosis represents a discordant finding. Potential etiologies for this include secondary erythrocytosis related to chronic hypoxia, renal disease with erythropoietin dysregulation, or another unrecognized myeloproliferative neoplasm. For this patient, a formal evaluation of Jak2 mutation status, serum EPO levels, and other hypoxia-related causes was not completed, which could represent an additional unresolved diagnostic variable.

In addition, he presented with low to mildly decreased calcium levels rather than the typical hypercalcemia you would expect. While you would also expect renal impairment, the patient's findings were consistent with his known chronic kidney disease rather than an acute myeloma-related renal failure. The CRAB criteria represent a descriptive clinical framework for multiple myeloma and do not constitute a proprietary or licensed diagnostic tool. Finally, he had no detectable monoclonal protein on electrophoresis and a negative urine immunofixation electrophoresis. 

Given these contraindicatory findings, one of the most intriguing aspects of this case was the markedly elevated serum-free kappa light chain levels and the abnormal kappa-to-lambda ratio. Serum free light chain testing is especially useful for identifying light chain-only multiple myeloma, a condition in which SPEP may be negative. Chronic kidney disease may result in reduced clearance of free light chains, particularly kappa chains, leading to elevated levels and an increased kappa-to-lambda ratio in the absence of plasma cell malignancy.

However, interpretation of free light chain assays can be complicated in patients with impaired renal function [5]. Chronic kidney disease can lead to reduced clearance of light chains, often resulting in elevated absolute values and distorted ratios even in the absence of plasma cell malignancy [5]. The kappa-to-lambda ratio of 11.35, while concerning, falls within a range observed in nonmalignant renal disease, particularly when interpreted in isolation.

A reduced glomerular filtration rate (GFR) is known to impair renal clearance of Kappa free light chains disproportionately relative to Lambda free light chains. This results in the elevation of the Kappa Lambda ratio, independent of monoclonal plasma cell proliferation. In patients with chronic kidney disease, an adjusted estimated GFR (eGFR) reference range for free light chain ratios has been proposed, with the upper limits exceeding those compared to individuals who have preserved renal function. In this patient with an eGFR of around 50 mL/min, the Kappa Lambda ratio of 11.35 must therefore be interpreted cautiously since the renal dysfunction may play a role in reducing the specificity of the free light chain abnormalities for clonal disease. Consequently, the elevated ratio alone cannot be used as definitive evidence of monoclonality in the absence of marrow infiltration, immune fixation, or lesion-directed histopathology.

The negative CT-guided bone biopsy further complicated the diagnostic picture. However, sampling from the iliac crest may not reflect focal disease at other skeletal sites due to the patchy nature of marrow involvement. Iliac crest bone marrow biopsy remains the gold standard for diagnosing plasma cell dyscrasias; however, marrow involvement in multiple myeloma is often heterogeneous and can be focal [11,12]. False-negative biopsies have been well-described in the literature, particularly in early disease or when lytic lesions are distant from the biopsy site [13]. Therefore, a single negative biopsy cannot definitively exclude myeloma or other infiltrative marrow processes and is not representative of the lesions seen in the skull or spine. Given the patient's clinical status and prior neurological medical history, further investigation was not done immediately at the time.

Alternative explanations for diffuse lytic lesions should also be considered. Metastatic disease from solid tumors such as lung, breast, renal, or prostate cancer can produce osteosclerotic lesions as well [14]. These lesions can present similar imaging findings, though this patient’s cross-sectional imaging and tumor markers failed to identify a primary malignancy. Additionally, nonmalignant conditions, such as metabolic disease, benign bone cysts, and degenerative changes, may, on occasion, mimic lytic lesions but are less likely to produce the diffuse, multifocal pattern observed in this case [15].

Other alternatives for osteolytic lesions include rare cases of monoclonal gammopathy of undetermined significance (MGUS) or smoldering myeloma with solitary or multifocal lytic lesions, which have been reported in the absence of the classic end-organ damage that is usually seen. These differentials occupy a diagnostic gray zone in the realm of plasma cell disorders and highlight the limitations of rigid dichotomization between malignant and benign diseases and their states of presentation. In this particular case, the absence of a definitive bone marrow biopsy with the involvement of an incomplete monoclonal protein characterization leaves the possibility of an unresolved early or borderline plasma cell disorder.

Regarding this patient's treatment, his clinical course favored a conservative approach. He denied any bone pain, constitutional symptoms, or progressive functional decline apart from his one episode, and his gait instability was more plausibly explained by the prior cerebral vascular disease rather than spinal cord compression or malignant infiltration based on radiographic findings. In the absence of a definitive diagnostic criterion for multiple myeloma or metastatic disease, oncologists appropriately recommended closer surveillance rather than aggressive or empirical therapy. The patient continues to see his neurologist, for close follow-up of symptoms of peripheral weakness and routine care following his previous cerebrovascular accident (CVA), as well as his cardiologist.

## Conclusions

This unique case highlights the limitations of relying solely on diagnostic criteria. It shows the diagnostic complexity encountered when imaging shows potential malignant bone lesions, but laboratory and histopathological findings are inconclusive. Diffuse lytic bone lesions in the setting of an abnormal serum free light chain ratio is concerning for plasma cell dyscrasias. The absence of classic findings, such as anemia, hypercalcemia, monoclonal protein on electrophoresis, and a positive bone marrow biopsy, underscores the limitations, especially in patients with chronic kidney disease, and reinforces the role of longitudinal follow-up in managing diagnostic uncertainty. It highlights the importance of renal-adjusted biomarker interpretation, lesion-directed biopsy when feasible, and surveillance as the next clinical step in patients with suspected plasma cell dyscrasias that are potentially inconclusive on initial evaluation.
